# Single‐dose of LC51‐0255, a selective S1P_1_ receptor modulator, showed dose‐dependent and reversible reduction of absolute lymphocyte count in humans

**DOI:** 10.1111/cts.13227

**Published:** 2022-01-23

**Authors:** Sang Won Lee, Inyoung Hwang, Jaeseong Oh, SeungHwan Lee, In‐Jin Jang, Kyung‐Sang Yu

**Affiliations:** ^1^ Department of Clinical Pharmacology and Therapeutics Hanyang University Seoul Hospital Seoul South Korea; ^2^ Department of Clinical Pharmacology and Therapeutics Seoul National University College of Medicine and Hospital Seoul South Korea

## Abstract

Reducing the peripheral absolute lymphocyte count (ALC) is a promising therapeutic approach in treating autoimmune diseases. LC51‐0255 is a sphingosine‐1‐phosphate 1 receptor modulator, which is known to decrease the peripheral ALC. We aimed to assess the pharmacokinetics (PKs), pharmacodynamics (PDs), safety, and tolerability profiles of LC51‐0255 after a single oral administration in healthy subjects. A randomized, double‐blind, placebo‐controlled, dose‐escalation study was conducted in 50 healthy subjects. Each subject orally received LC51‐0255 (0.25, 0.5, 1, 2, or 4 mg) or its matching placebo in an 8:2 ratio. Blood and urine samples were collected to assess the PKs, and PDs was evaluated using peripheral ALC and 24‐h hourly heart rate data. Safety and tolerability were assessed by monitoring treatment emergent adverse events (TEAEs), vital signs, 12‐lead electrocardiogram (ECG), continuous 24‐h ECG (via Holter monitoring), clinical laboratory tests, ophthalmologic tests, pulmonary function tests, and physical examinations. A single dose of LC51‐0255 reduced ALC and heart rate in a reversible and dose‐dependent manner. Systemic exposure of LC51‐0255 increased dose‐dependently and its half‐life ranged from 72.2 to 134.0 h. ALC and the systemic exposure of LC51‐0255 seemed to be negatively correlated. LC51‐0255 was well‐tolerated up to 2 mg, and the most common TEAE was bradycardia. The results of this study suggest that LC51‐0255 can be developed into a beneficial treatment option for autoimmune disease.

**Study Highlights**

**WHAT IS THE CURRENT KNOWLEDGE ON THE TOPIC?**

Reducing the peripheral absolute lymphocyte count (ALC) is a promising therapeutic approach to treat autoimmune diseases. Sphingosine‐1‐phosphate 1 (S1P_1_) receptor modulator reduces peripheral ALC by preventing the recirculation of lymphocytes from lymphatic tissue to target organs.

**WHAT QUESTION DID THIS STUDY ADDRESS?**

We performed this study to assess the pharmacokinetics, pharmacodynamics, safety, and tolerability profiles of LC51‐0255, a novel S1P_1_ receptor modulator, in humans.

**WHAT DOES THIS STUDY ADD TO OUR KNOWLEDGE?**

Our results showed that LC51‐0255 has a relatively long half‐life, is well‐tolerated, and reduces ALC in a dose‐dependent and reversible manner.

**HOW MIGHT THIS CHANGE CLINICAL PHARMACOLOGY OR TRANSLATIONAL SCIENCE?**

Our results provide evidence that a single dose of LC51‐0255 can be further developed into a beneficial treatment option for patients with autoimmune disease.

## INTRODUCTION

Autoimmune diseases, such as ulcerative colitis, Crohn’s disease, and multiple sclerosis can cause life‐long morbidity and disability. It has been reported that up to 3–5% of the general population suffers from some form of autoimmune disease.^1^ Although autoimmune diseases can be caused by a variety of factors (e.g., host genes and environmental factors), they can be characterized by systemic or organ‐specific infiltration of immune cells (e.g., lymphocytes and macrophages).^2^, ^3^ Therefore, reducing the peripheral absolute lymphocyte count (ALC) to attenuate aberrant immune responses is considered a promising therapeutic approach to treat autoimmune diseases.^4^, ^5^

Sphingosine‐1‐phosphate receptors (S1P_1_–S1P_5_) are subtypes of G protein‐coupled receptor family, and they are known to be responsible for variety of cellular response (e.g., immune cell trafficking, vascular homeostasis, and heart rate reduction), which has been extensively reviewed by many researchers.^6^, ^7^, ^8^ Fingolimod is the first sphingosine‐1‐phosphate (S1P) receptor modulator that was approved by the US Food and Drug Administration as a treatment for relapsing form of multiple sclerosis (MS) in 2010. After the success of fingolimod, next generation S1P receptor modulators (e.g., ponesimod, ozanimod, and siponimod) are being developed. Based on the fact that fingolimod is a nonselective S1P receptor (S1P_1_, S1P_3_, S1P_4_, and S1P_5_) modulator, whereas its lymphocyte lowering effect is believed to be mediated by modulating S1P_1_ receptors,^9^ the main strategy for next generation S1P receptor modulators have been improving the receptor selectivity in order to reduce undesired effects. For example, preclinical studies in mice have suggested that S1P_3_ may be related to undesired cardiovascular effects.^10^ However, little is known about adverse events specifically driven by S1P_4_ and S1P_5_.

Heart rate (HR) reduction after first‐dose administration is a known side effect of S1P receptor modulators.^11^, ^12^, ^13^, ^14^ The reduced HR is caused by S1P receptor dependent activation of G‐protein‐coupled inwardly rectifying potassium channels on atrial myocytes, which results in temporary reduction in excitability.^15^ Studies on mice suggested that the undesired cardiovascular effect may be induced by S1P_3_ modulation.^10^ However, there seems to be a species distinction regarding the role of S1P_3_ in cardiovascular effects because clinical studies with selective S1P receptor modulators repeatedly have shown that reducing the activity on S1P_3_ did not eliminate bradycardia in humans.^12^, ^13^, ^14^

LC51‐0255 (LG Chem) is a potent, selective, and orally available S1P_1_ receptor modulator that reduces peripheral ALC by preventing the recirculation of lymphocytes from lymphatic tissue to target organs. Once LC51‐0255 is bound to the S1P_1_ receptor, it is internalized from the cell membrane and is introduced to the receptor degradation pathway, leading to the capture of immune cells in the secondary lymph node. Single daily oral administration of LC51‐0255 at 0.03, 0.1, 0.3, and 1 mg/kg in rats resulted in a dose‐dependent reduction in peripheral ALC, particularly T‐cells.^16^ The human equivalent doses (HEDs) for 0.03, 0.1, 0.3, and 1 mg/kg in rats are 0.0048, 0.016, 0.048, and 0.16 mg/kg, which can be translated into 0.29, 0.96, 2.88, and 9.60 mg for 60 kg adult human. Additionally, the no observed adverse event levels of LC51‐0255 in rats and monkeys were both 3 mg/kg, which can be translated into 2.88 and 5.76 mg for 60 kg adult human, respectively.^16^ Among these HEDs in preclinical efficacy and safety results, the lowest dose was 0.29 mg assuming 60 kg adult human, which was the basis of the starting dose of this study (i.e., 0.25 mg).

Based on the preclinical findings, we believe that LC51‐0255 could be developed as a therapeutic option for patients with autoimmune diseases. Therefore, we conducted a phase I study to assess the pharmacokinetics (PKs), pharmacodynamics (PDs), safety, and tolerability profiles of LC51‐0255 after a single administration in humans.

## METHODS

### Subjects

Healthy Korean men aged 19–45 years with a body mass index of 18–27.0 kg/m^2^ were enrolled in this study. Eligibility was assessed based on medical history, physical examination, vital signs, 12‐lead electrocardiogram (ECG), clinical laboratory test, serology (i.e., hepatitis B, hepatitis C, human immunodeficiency virus, syphilis, and tuberculosis), chest radiography, pulmonary function tests, ophthalmologic tests, and urinary drug screening. Subjects with absolute white blood cell counts lower than 3500 cells/μl or lymphocyte counts lower than 800 cells/μl were excluded from this study. This study was approved by the Institutional Review Board of the Seoul National University Hospital and conducted in accordance with the principles of the Declaration of Helsinki and Korean Good Clinical Practice (www.clinicaltrials.gov registration number: NCT03174613). For every subject, written informed consent was obtained before any study‐related procedures were performed.

### Study design

This was a randomized, double‐blind, placebo‐controlled, dose‐escalation phase I clinical study of LC51‐0255, with a single oral administration of 0.25, 0.5, 1, 2, or 4 mg in healthy subjects. The 2 mg dose group was conducted as a fixed sequence, two‐period, crossover study to evaluate the effect of food on the systemic exposure of LC51‐0255. Therefore, subjects were given 2 mg LC51‐0255 (or placebo) either in fasted or fed (high‐fat meal 30 min before LC51‐0255 administration; 900 kcal or more; fat content 40% or more) conditions, with a washout period of at least 21 days. Eligible subjects were admitted to the Seoul National University Hospital Clinical Trial Center 2 days prior to dosing (day −2) and discharged 7 days after dosing (day 8). Baseline hourly mean heart rates (Holter monitoring) were obtained 1 day before dosing (day −1). Each dose group included 10 subjects, who randomly received LC51‐0255 or placebo in a ratio of 8:2.

### Pharmacokinetic analysis

PKs were evaluated using plasma and urine LC51‐0255 concentrations. Serial blood samples (5 ml per sample) were collected in sodium heparin tubes at 0 (predose), 1, 2, 3, 4, 5, 6, 7, 8, 10, 12, 24, 36, 48, 72, 96, 120, 144, and 168 h postdose. The samples were centrifuged within 30 min, and two aliquots (1 ml each) were stored below −70°C until analysis. In higher dose groups (i.e., 2 and 4 mg), additional blood samples were collected (2 mg: 216, 264, and 312 h postdose in each period; 4 mg: 216, 264, 312, 360, 408, 456, and 504 h postdose) to determine whether the log‐linear slope was maintained. Urine samples were collected up to 168 h postdose. Two aliquots (1 ml each) per time interval (0–24, 24–48, 48–72, 72–96, 96–120, 120–144, and 144–168 h postdose) were stored below −70°C until analysis.

PK parameters were derived with a noncompartmental method using Phoenix WinNonlin software (version 8.1; Certara USA, Inc.). The maximum plasma concentration (C_max_) and time to reach C_max_ (T_max_) were obtained directly from the observed values. The area under the plasma concentration‐time curve (AUC) from time zero to the last measurable time point (AUC_last_) was determined using the linear trapezoidal method for ascending concentrations and the log‐trapezoidal method for descending concentrations. The AUC from time zero to infinity (AUC_inf_) was calculated as AUC_last_ + Ct/λ_z_, where C_t_ is the last measurable concentration and λ_z_ is the elimination rate constant estimated from linear regression of the log‐linear portion of the plasma concentration‐time curve. AUC_0–168 h_ was defined as the area under the plasma concentration‐time curve from time zero to 168 h postdose, whereas AUC extrapolated (AUC_extrap_) was defined as Ct/λ_z_ divided by AUC_inf_. The terminal half‐life (t_1/2_) is the natural logarithm of 2 divided by λ_z_. The apparent clearance was the dose divided by the AUC_inf_. For urinary PK parameters, amount of unchanged LC51‐0255 excreted in urine, fraction of unchanged LC51‐0255 excreted in urine, and renal clearance (CL_R_ = A_e_/AUC_inf_) was calculated.

### Pharmacodynamic analysis

PDs were evaluated using ALC in the peripheral blood, obtained at (day 1) 0 h (predose), 2, 4, 6, 8, 12, 24, 36, 48, 60, 72, 96, 120, 144, and 168 h postdose. In the higher dose groups (2 and 4 mg), ALC was observed at additional time points to determine if ALC recovered to baseline level (2 mg: up to 312 h postdose, 4 mg: up to 504 h postdose) after dosing. PD parameters including ALC at maximum effect (E_max_), change from baseline in absolute lymphocyte count at maximum effect (ΔE_max_), area under the absolute lymphocyte count‐time curve from time zero to 168 h postdose (AUEC_0–168 h_), baseline corrected area under the absolute lymphocyte count‐time curve from time zero to 168 h postdose (ΔAUEC_0–168 h_), time to reach maximum effect (TE_max_), and percent change from baseline in absolute lymphocyte count at maximum effect (CFB_max_), were acquired using the Phoenix WinNonlin software (version 8.1; Certara USA, Inc.). Baseline was defined as the value measured at 1 day 0 h (predose) for each subject.

Additionally, 24‐h hourly HRs were also observed for the PD evaluation. To do so, baseline 24‐h hourly mean HRs were obtained 1 day before dosing (day −1) and compared with 24‐h hourly mean heart rates obtained after dosing (day 1) via Holter monitoring.

### Determination of plasma and urine Concentration of LC51‐0255

Plasma and urine LC51‐0255 concentrations were determined using a fully validated liquid chromatography‐tandem mass spectrometry (LC‐MS/MS) method operated in positive mode by International Science Standards, Inc. To measure plasma LC51‐0255 concentrations, 300 μl of acetonitrile was added to 100 μl of the plasma sample, and 200 μl of 50% acetonitrile was added to 50 μl of the urine sample. The samples were then vortexed and centrifuged. A portion of the supernatant was then transferred to autosampler vials for injection into the LC‐MS/MS system. Chromatographic retention of LC51‐0255 was obtained on a Luna Phenyl‐Hexyl, 2.0 × 100 mm, 3 μm particle size column (Phenomenex) under gradient conditions with a flow rate of 0.3 ml/min. LC51‐0255 was detected by multiple reaction monitoring using an MDS SCIEX API 5000 (API 4000 for urine) mass spectrometer (Applied Biosystems/MDS SCIEX) in electrospray ionization in positive mode. The mass transitions monitored for LC51‐0255 were m/z 528.247 → 207.100. The methods were validated over the assay range of 0.3 to 1000 ng/ml for plasma concentrations and 0.3 to 300 ng/ml for urine concentrations. The average regression correlation coefficients (r) for plasma and urine were greater than 0.9985 and greater than 0.9993, respectively. The range of accuracies for plasma and urine were 95.9%–93.9% and 97.9%–93.9%, respectively. The lower limit of quantification was 0.3 ng/ml for both plasma and urine. Based on the observed performance of the assays, the results of the human plasma and urine samples reported in this study were accurate and reliable.

### Safety and tolerability assessment

Safety and tolerability were assessed by monitoring treatment emergent adverse events (TEAEs), vital signs, 12‐lead ECG, continuous 24‐h ECG (via Holter monitoring), clinical laboratory tests, ophthalmologic tests, pulmonary function tests (FVC, FEV1, and FEV/FVC), and physical examinations. Adverse events of special interest included bradycardia (defined as <40 bpm), high‐degree atrioventricular (AV) blocks, decreased pulmonary function, and macular edema. TEAEs were classified according to the Medical Dictionary for Regulatory Activities (version 20.0), and the investigators reviewed the results on the relationship to LC51‐0255 and clinical significance.

### Statistical analysis

Demographic characteristics, PK, and PD parameters were summarized using descriptive statistics. The Kruskal‐Wallis test was used to compare the demographic characteristics and PD parameters between the treatment groups. The Pearson correlation coefficient was calculated to assess the PK‐PD relationship. We considered the PK to be dose‐proportional if the 95% confidence interval (CI) of the slope of the regression line for log‐transformed C_max_, AUC_0–168 h_, and AUC_inf_ using the power model included 1. We concluded that food had no significant effect on the bioavailability of LC51‐0255 if the geometric mean ratio (GMR) and its 90% CI of the fed condition to fasted condition for the log‐transformed C_max_ and AUC_last_ fell within the predefined range of 0.80–1.25. Statistical significance was set at *p* < 0.05. All analyses were performed using the SAS software (version 9.4; SAS Institute).

## RESULTS

### Subjects

All enrolled subjects were healthy Korean men in this study. One subject dropped out before study drug administration (due to failing to provide predose urine sample) and was replaced by another subject; therefore, a total of 51 subjects were enrolled in this study. As a result, 50 subjects completed the study, and their demographic characteristics were comparable among the treatment groups (*p* > 0.05). The mean ± standard deviation of age, weight, height, and body mass index were 30.5 ± 5.5 years, 70.0 ± 8.0 kg, 1.74 ± 0.05 m, and 23.2 ± 2.0 kg/m^2^, respectively.

### Pharmacokinetics

**FIGURE 1:**
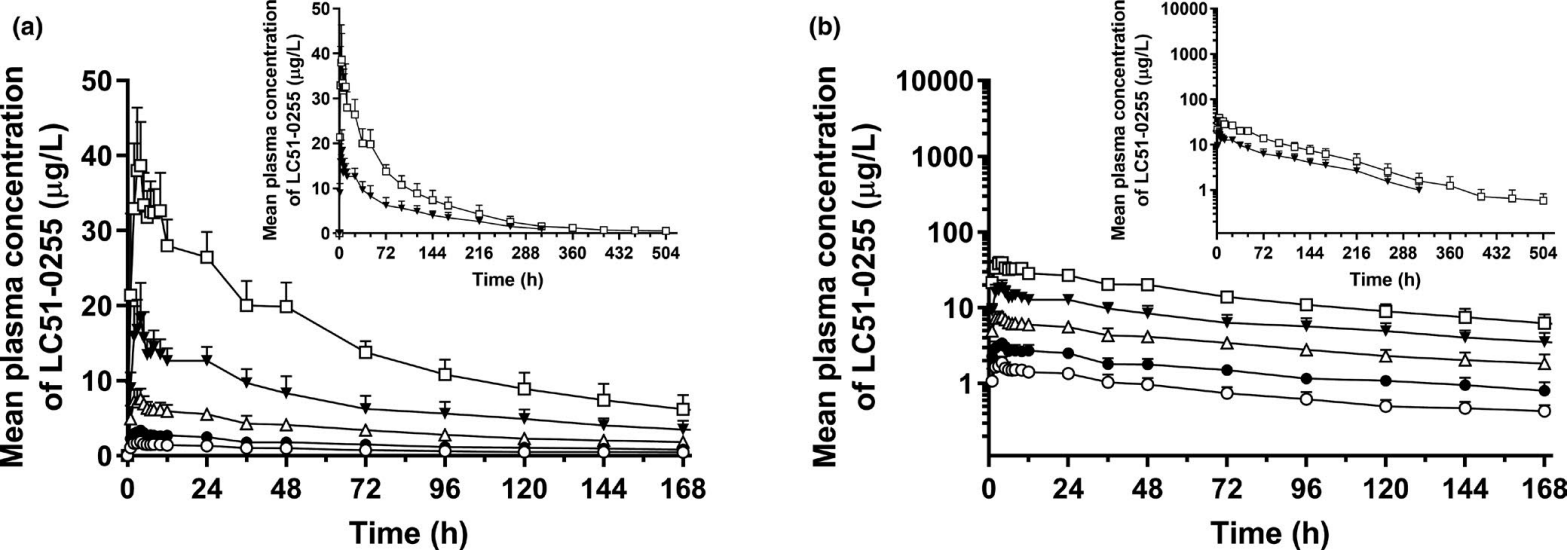
Mean plasma concentration‐time profiles of LC51‐0255 after a single oral administration of LC51‐0255 up to 168 h postdose (insets: up to 312 h postdose for 2 mg dose group; up to 504 h postdose for 4 mg dose group) in healthy male subjects. (a) Linear scale. (b) Semi‐log scale. The error bars denote the standard deviations (○ = 0.25 mg, *N* = 8; ● = 0.5 mg, *N* = 8; △ = 1 mg, *N* = 8; ▼ = 2 mg, *N* = 8; □ = 4 mg, *N* = 8)

Systemic exposure to LC51‐0255 increased dose‐dependently after a single dose, with the mean AUC ranging from 130 to 2500 μg/L (Table 1). The peak plasma concentration of LC51‐0255 was observed at 3–4 h. LC51‐0255 was slowly removed (mean t_1/2_: 72.2–134.0 h) from the body, in such a rate that it was still detectable 504 h postdose in the 4 mg dose group (Figure 1). The fraction excreted into urine was less than 0.1% across all dose levels, indicating that LC51‐0255 was mainly eliminated by non‐renal pathways. LC51‐0255 showed slightly more than dose‐proportional exposure (within 20%), because the 95% CI of slope of regression for log‐transformed C_max_, AUC_0–168 h_, and AUC_inf_ were 1.07–1.18, 1.02–1.13, and 0.97–1.12, respectively. The GMR (90% CI) of the fed condition to fasted condition for AUC_last_ and AUC_inf_ were 0.984 (0.929–1.040) and 1.009 (0.920–1.105), respectively, which fell within the predefined bioequivalence range, whereas that of C_max_ did not (0.831 [0.749–0.922]).

**TABLE 1:** Pharmacokinetic parameters of LC51‐0255 after a single oral administration in healthy male subjects

PK Parameters	0.25 mg	0.5 mg	1 mg	2 mg	4 mg
	(*N* = 8)	(*N* = 8)	(*N* = 8)	(*N* = 8)	(*N* = 8)
T_max_, h	4.00 (2.00−4.05)	2.52 (1.00−5.00)	4.00 (2.00−5.00)	4.00 (4.00−8.00)	3.50 (3.00−8.00)
C_max_, μg/L	1.88 ± 0.37	3.44 ± 0.45	8.15 ± 1.05	19.30 ± 3.02	39.70 ± 6.36
AUC_0–168 h_, h·μg/L	130.0 ± 25.6	251.0 ± 30.4	570.0 ± 79.0	1190.0 ± 235.0	2500.0 ± 379.0
AUC_inf_, h·μg/L	188.0 ± 54.8	341.0 ± 39.7	945.0 ± 279.0	1610.0 ± 311.0	3200.0 ± 700.0
AUC_extrap_, %	30.6 ± 8.2	26.6 ± 3.7	36.2 ± 15.3	6.7 ± 4.1	1.7 ± 0.6
t_1/2_, h	100.0 ± 45.1	87.3 ± 9.0	134.0 ± 65.4	72.2 ± 30.9	76.3 ± 15.4
CL/F, L/h	1.4 ± 0.4	1.5 ± 0.2	1.1 ± 0.3	1.3 ± 0.2	1.3 ± 0.3
V_d_/F, L	192.0 ± 48.0	186.0 ± 22.3	200.0 ± 66.2	130.0 ± 50.2	139.0 ± 23.9
A_e_, µg	0	0	0.83 ± 2.01	0.19 ± 0.54	0.34 ± 0.58
F_e_, %	0	0	0.08 ± 0.20	0.01 ± 0.03	0.01 ± 0.01
CL_R_, ml/h	0	0	0.66 ± 1.47	0.12 ± 0.33	0.11 ± 0.21

Note

All data are presented as mean ± standard deviation, except for T_max_, which is presented as the median (minimum‐maximum).

Abbreviations: A_e_, amount of unchanged LC51‐0255 excreted in urine; AUC_0–168 h_, area under the plasma concentration‐time curve from time 0 to 168 h post‐dose; AUC_extrap_, area under the plasma concentration‐time curve extrapolated; AUC_inf_, area under the plasma concentration‐time curve from time zero to infinity; C_max_, peak plasma concentration; CL/F, apparent clearance; CL_R_, renal clearance (A_e_/AUC_inf_); F_e_, fraction of unchanged LC51‐0255 excreted in urine; t_1/2_, terminal half‐life; PK, pharmacokinetic; T_max_, time to reach peak plasma concentration; V_d_/F, apparent volume of distribution.

### Pharmacodynamics

**FIGURE 2:**
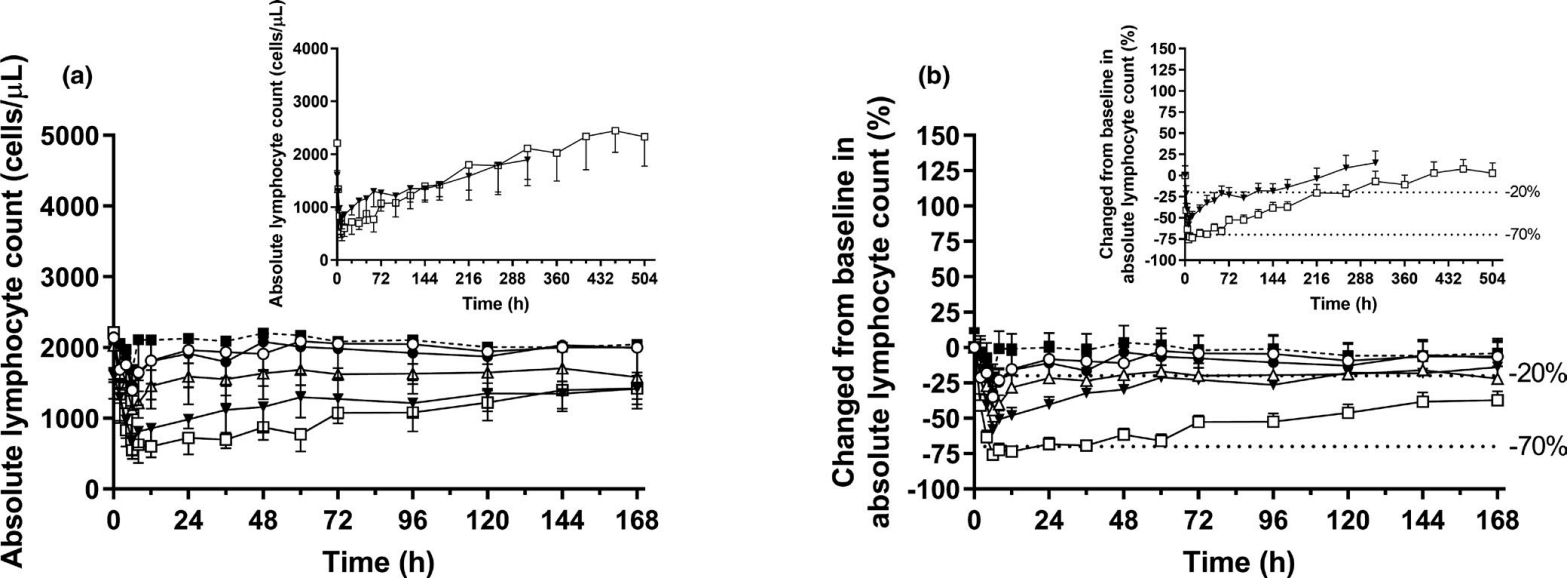
Time course of absolute lymphocyte count after a single oral administration of LC51‐0255 (or placebo) up to 168 h post‐dose (insets: up to 312 h post‐dose for 2 mg dose group; up to 504 h post‐dose for 4 mg dose group) in healthy male subjects. (a) Mean absolute lymphocyte count. (b) Percent change from baseline in absolute lymphocyte count. The error bars denote the standard deviations. Upper dotted line: −20% change from baseline, related to minimal efficacy^19^, ^20^; lower dotted line: −70% change from baseline, related to plateau of efficacy^19^, ^20^ (○ = 0.25 mg, *N* = 8; ● = 0.5 mg, *N* = 8; △ = 1 mg, *N* = 8; ▼ = 2 mg, *N* = 8; □ = 4 mg, *N* = 8; ■ = placebo, *N* = 10)

A single administration of LC51‐0255 resulted in a dose‐dependent and reversible reduction in ALC (Figure 2 and Figure S1). LC51‐0255 reached its maximum PD effect within 6 to 10 h postdose at all dose levels, with the maximum percent change from baseline of ALC ranging from −34% to −77% (Table 2, Figure 2). LC51‐0255 decreased E_max_ and AUEC_0–168 h_ significantly in 1, 2, and 4 mg dose groups in a dose‐dependent manner compared to the placebo group (E_max_: 1585, 1058, 598, and 488 cells/µl for placebo, 1, 2, and 4 mg, respectively; area under the effect curve [AUEC]_0–168 h_: 348,612, 271,210, 205,505, and 176,470 h·cells/μl for placebo, 1, 2, and 4 mg, respectively). Moreover, the changes from baseline in ALC (ΔE_max_ and ΔAUEC_0–168 h_) due to LC51‐0255 were particularly pronounced in the 4 mg dose group (Table 2, Figure S1). The decreased ALC levels recovered to the normal range (>1000 cells/ml) within 96 h postdose (Figure 2).

**TABLE 2:** Pharmacodynamic parameters of LC51‐0255 after a single oral administration in healthy male subjects

PD Parameters	0.25 mg	0.5 mg	1 mg	2 mg	4 mg	Placebo
	(*N* = 8)	(*N* = 8)	(*N* = 8)	(*N* = 8)	(*N* = 8)	(*N* = 10)
TE_max_, h	6.00 (2.00–36.00)	6.00 (6.00–35.98)	6.00 (6.00–8.00)	6.00 (6.00–36.00)	10.00 (6.00–60.00)	6.00 (0–168.00)
E_max_, cells/µl	1358 ± 230	1406 ± 331	1058 ± 223*	598 ± 102**	488 ± 99**	1585 ± 235
ΔE_max_, cells/μl	−784 ± 611	−744 ± 311	−971 ± 503	−1050 ± 346	−1780 ± 579**	−543 ± 541
AUEC_0–168 h_, h·cells/μl	331005 ± 62641	325604 ± 64881	271210 ± 41834*	205505 ± 32288**	176470 ± 29175**	348612 ± 50167
ΔAUEC_0–168 h_, h·cells/μl	−28757 ± 57540	−35605 ± 30802	−69603 ± 64364	−71273 ± 38679	−204451 ± 78422**	−8712 ± 83989
CFB_max_, %	−33.52 ± 17.92	−33.78 ± 12.34	−45.42 ± 14.55	−62.58 ± 8.63	−76.87 ± 9.25	−21.98 ± 17.27

Note: All data are presented as mean ± standard deviation, except for TE_max_, which is presented as the median (minimum‐maximum).

Abbreviations: ΔAUEC_0–168 h_, baseline corrected area under the absolute lymphocyte count‐time curve from time zero to 168 h post‐dose; ΔE_max_, change from baseline in absolute lymphocyte count at maximum effect; AUEC_0–168 h_, area under the absolute lymphocyte count‐time curve from time zero to 168 h post‐dose; CFB_max_, percent change from baseline in absolute lymphocyte count at maximum effect; E_max_, absolute lymphocyte count at maximum effect; TE_max_, time to reach maximum effect; PD, pharmacodynamic.

*p value <0.05 vs. placebo; ^**^ *p* value <0.0001 vs. placebo.

**FIGURE 3:**
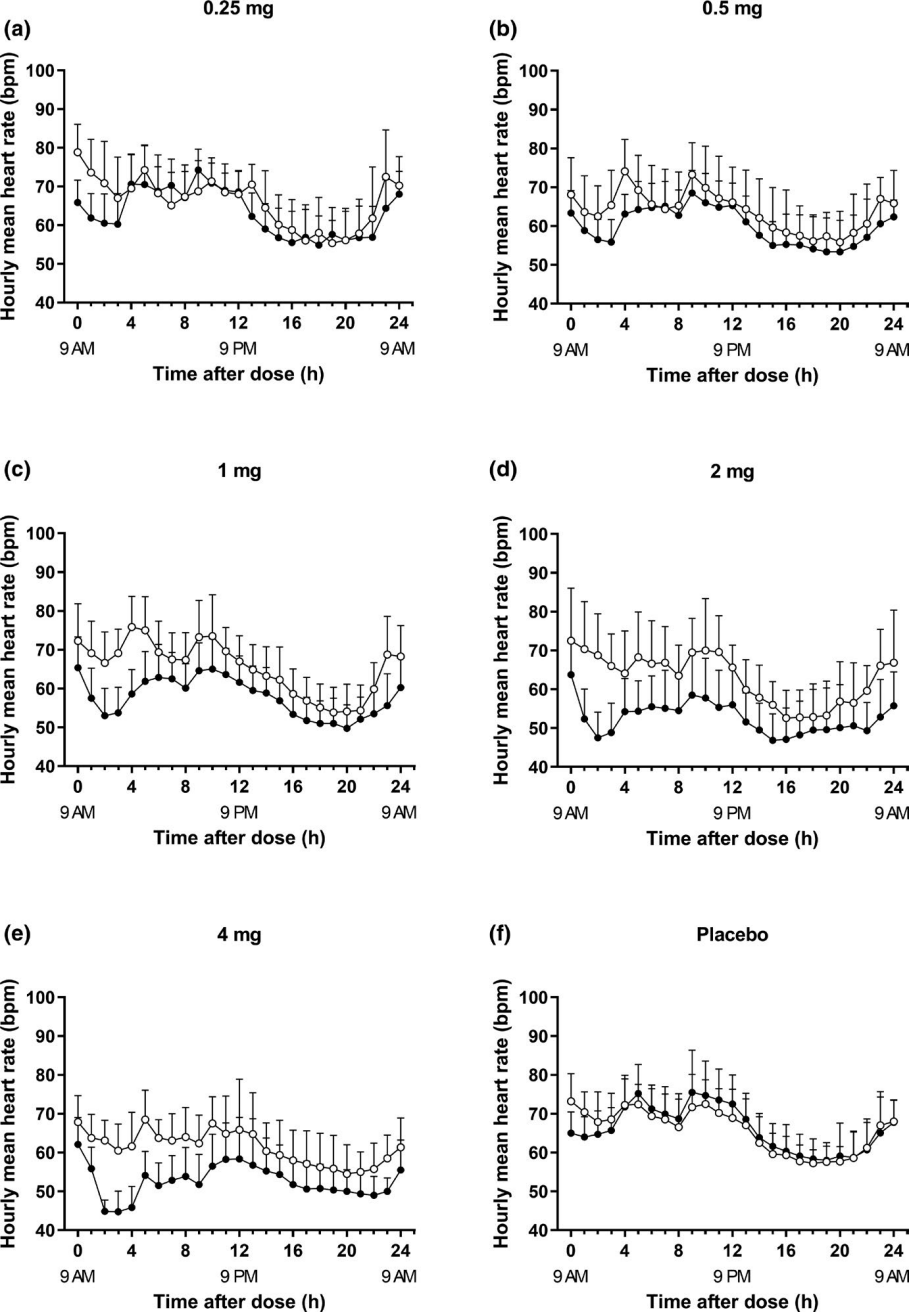
Mean hourly heart rate‐time profiles after a single oral administration of LC51‐0255 (or placebo) up to 24 h postdose in healthy male subjects. (a) 0.25 mg (*N* = 8); (b) 0.5 mg (*N* = 8); (c) 1 mg (*N* = 8); (d) 2 mg (*N* = 8); (e) 4 mg (*N* = 8); (f) placebo (*N* = 10). The error bars denote the standard deviations (○ = baseline, ● = day 1)

As expected, a dose‐dependent, transient decrease in HR was observed in all dose groups (Figure 3). The bradycardia criterium for this study was 40 bpm, and mean hourly HRs returned to levels above 50 bpm 5 h after dosing. The decrease in hourly mean HRs 12 h postdose was presumed to be a physiological decrease due to increased vagal tone during resting hours.

### PK‐PD relationship

**FIGURE 4:**
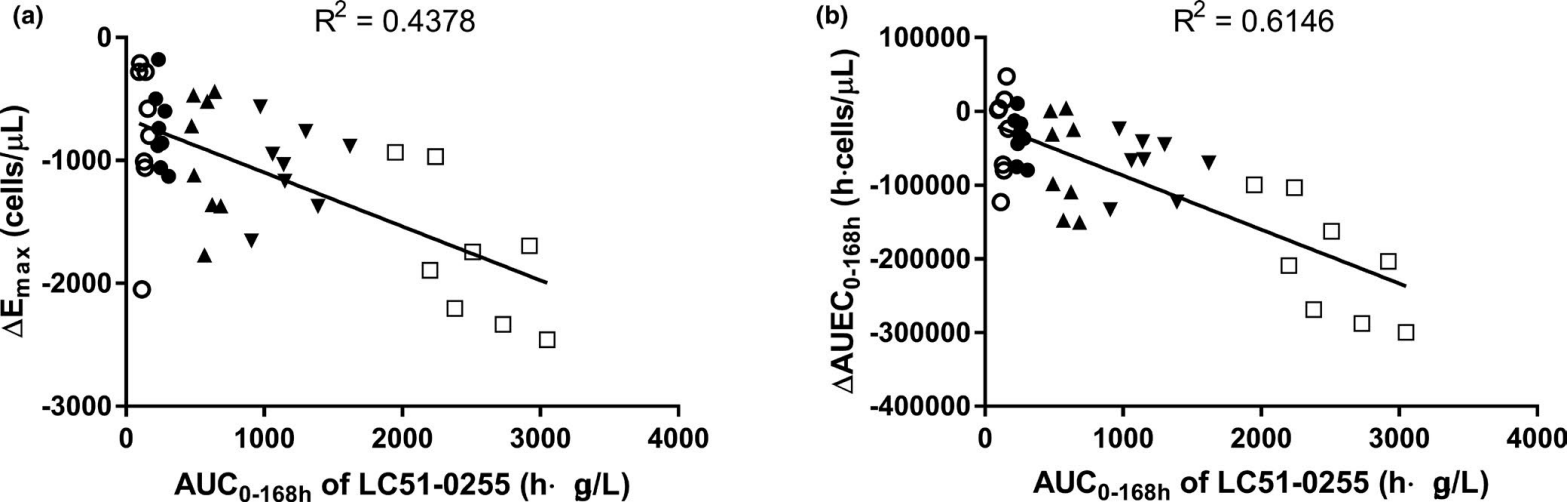
Relationship between individual pharmacokinetic parameters versus pharmacodynamic parameters after a single oral administration of LC51‐0255 in healthy male subjects. (a) AUC_0–168 h_ versus ΔE_max_; (b) AUC_0–168 h_ versus ΔAUEC_0–168 h_. (○ = 0.25 mg, *N* = 8; ● = 0.5 mg, *N* = 8; △ = 1 mg, *N* = 8; ▼ = 2 mg, *N* = 8; □ = 4 mg, *N* = 8). AUC_0–168 h_, area under the plasma concentration‐time curve from time zero to 168 h postdose; ΔAUEC_0–168 h_, area under the absolute lymphocyte count‐time curve from time zero to 168 h postdose

ALC (i.e., ΔE_max_ and ΔAUEC_0–168 h_) was negatively correlated (*p* < 0.0001) with the systemic exposure of LC51‐0255 (i.e., C_max_ and AUC_0–168 h_), particularly between ΔAUEC_0–168 h_ and AUC_0–168 h_ (*R* ^2^ = 0.6146; Figure 4, Figure S2). Results also showed that the ALC change from baseline was negatively correlated (*p* < 0.0001) with plasma concentration of LC51‐0255 (Figure S3).

### Safety and tolerability

LC51‐0255 was well‐tolerated after a single oral administration of up to 2 mg. A total of 33 TEAEs occurred in 13 subjects (32.5%); however, there were no serious adverse events in this study. All TEAEs, except for one case (dental caries), resolved without any treatment. The most common TEAE, bradycardia (10 subjects), occurred more frequently in the higher dose groups (2 and 4 mg; Table S1). In the 4 mg dose group, one case of asymptomatic second degree atrioventricular block with bradycardia was observed, which resolved spontaneously within a day. No other clinically significant abnormalities or changes were observed in the ECG (including QT prolongation), Holter monitoring, vital signs, physical examination, clinical laboratory tests, pulmonary function tests, and ophthalmologic tests.

## DISCUSSION

This study was a first‐in‐human study designed to evaluate the PDs, PKs, safety, and tolerability of LC51‐0255 in healthy humans. Intensive blood sampling and Holter monitoring allowed us to comprehensively evaluate the PD and PK characteristics of LC51‐0255.

LC51‐0255 exhibited a dose‐dependent increase in systemic exposure and a relatively long t_1/2_ compared to other S1P1 receptor modulators (t_1/2_ of ponesimod: 32 h; t_1/2_ of ozanimod: 19 h; t_1/2_ of siponimod: 30 h).^12^, ^13^, ^14^ The fact that LC51‐0255 has high apparent volume of distribution, S1P_1_ receptors are ubiquitously expressed in human,^17^ and bioavailability in rats was 80.7%^16^ may suggest an extensive distribution of LC51‐0255 in tissues. Food intake reduced the rate of absorption (i.e., C_max_) by ~ 17%, while showing no significant effect on the overall exposure of LC51‐0255 (i.e., AUC_last_ and AUC_inf_). Renal clearance was not an important elimination pathway, which was expected based on the fact that in rats, the amount of unchanged drug of LC51‐0255 excreted in urine was 0.52% and 0.13% for intravenous and oral administration, respectively.^16^

A single oral administration of LC51‐0255 decreased ALC in a dose‐dependent and reversible manner. It has been reported that the reduction of peripheral ALC is the result of the prevention of lymphocyte recirculation from lymphatic tissue to target organs,^18^ which implies that the ALC‐lowering effect of LC51‐0255 has little to do with the proliferation and differentiation of lymphocytes. This may explain the reversible nature of the ALC‐lowering effect of LC51‐0255 after a single administration. The reversible PD effect of LC51‐0255 could be beneficial in terms of minimizing the risk of infection.

Peak ALC reduction of 62%–70% from baseline is reported to be associated with a plateau of efficacy for treatment of MS, whereas an ~ 20% reduction is associated with minimal efficacy,^19^, ^20^ and the mean maximal ALC reduction from baseline after single oral administration of LC51‐0255 was −33.52% to −76.87%, which was comparable to other S1P_1_ receptor modulators.^13^, ^14^, ^21^ Therefore, considering the relatively long mean t_1/2_ of LC51‐0255 (72.2–134.0 h), the possibility that LC51‐0255 could reach sufficient PD effect even in lower dose groups (e.g., 0.25 and 0.5 mg) should be considered when conducting multiple‐dose studies in the future. Additionally, ~ 2‐h time delay between time to reach peak ALC reduction (TE_max_) and T_max_ was observed in all dose groups (Figure S4). This finding is in line with the fact that an indirect‐response maximum unbound systemic concentration (I_max_) model best described the effect of ponesimod on ALC,^22^, ^23^ and it may reflect the complex nature of S1P_1_ receptor mediated ALC reduction.

A transient decrease in HR is a well‐known effect of the S1P_1_ receptor modulator.^14^, ^21^ However, the S1P_1_ receptor modulator induced bradycardia is shown to disappear with repeated dosing by internalization of the S1P_1_ and/or S1P_3_ receptors expressed on cardiac cells.^12^ Additionally, a direct‐effect I_max_ model with tolerance development and circadian variation best described the relationship between ponesimod concentration and HR in a population PK/PD study.^24^ Based on these findings, S1P_1_ receptor modulators, such as ponesimod and ozanimod, have implemented up‐titration dosing regimen to minimize bradycardia,^13^, ^14^, ^25^ and the HR decrease at treatment initiation of ponesimod 2.5 mg and ozanimod 0.3 mg using up‐titration was similar to that of a single administration of LC51‐0255 0.5 mg (ponesimod up‐titration 2.5 mg: 8 bpm; ozanimod up‐titration 0.3 mg: 7 bpm; single administration of LC51‐0255 0.5 mg: 8 bpm).^13^, ^25^ Similar to fingolimod, which is initiated at its therapeutic dose, LC51‐0255 may not require an up‐titration regimen owing to a built‐in up‐titration based on a long t_1/2_. Interestingly, relationship between plasma concentration of LC51‐0255 and HR reduction exhibited a hysteresis‐like feature in all dose groups (Figure S5). This may be interpreted as an early sign of tolerance development, and it would be interesting to investigate whether the negative chronotropic effect of LC51‐0255 also attenuates with repeated dosing in future studies.

Although TEAEs were more frequently reported with LC51‐0255 than with placebo, LC51‐0255 was relatively well‐tolerated after a single oral administration at doses ranging from 0.25 to 2 mg. However, we decided not to go beyond 4 mg in healthy volunteers because of the ample effect in reducing ALC seen with this dose and potential safety concerns at greater than 8 mg, such as cardiac dysrhythmia.

This study had a couple of major limitations. First, this was a single‐dose study conducted on healthy subjects. Therefore, the findings may not translate to patients with autoimmune diseases in a clinical setting. However, it has been repeatedly shown that the pharmacological effects of S1P_1_ receptor modulators in healthy subjects are in good agreement with those of patients with autoimmune diseases.^26^, ^27^, ^28^, ^29^, ^30^ Therefore, we believe our results should also translate well into patients with autoimmune diseases. Second, because only healthy male Koreans were enrolled in this study, the effect of sex and race on PKs, PDs, safety, and tolerability of LC51‐0255 could not be explored in this study. However, previous clinical studies that included both sexes and more than one race have shown no apparent effect of sex or race on PKs, PDs, safety, and tolerability of S1P modulators.^12^, ^28^, ^29^

In conclusion, this study showed that LC51‐0255 was generally well‐tolerated in healthy subjects after a single oral administration, and its PK and PD characteristics support the view that LC51‐0255 could be developed as a treatment option for patients with autoimmune disease.

## CONFLICT OF INTEREST

The authors declared no competing interest for this work.

## AUTHOR CONTRIBUTIONS

S.W.L. and K.‐S.Y. wrote the manuscript. All authors designed the research. All authors performed the research. S.W.L., I.H., and K.‐S.Y. analyzed the data.

## Supporting information

Figure S1

Figure S2

Figure S3

Figure S4

Figure S5

Table S1
