# Supplementary material for: Single‐dose of LC51‐0255, a selective S1P1 receptor modulator, showed dose‐dependent and reversible reduction of absolute lymphocyte count in humans
Source: Clin Transl Sci. 2022 Jan 23;15(4):1074–83. doi: 10.1111/cts.13227 (PMC9010277; doi:10.1111/cts.13227)

Figure S1. Pharmacodynamic parameters after a single oral administration of LC51-0255 (or placebo) in healthy male subjects. (A) E_{max}; (B) $AUEC_{0\text{-}168h}$; (C) ΔE_{max}; (D) $\Delta AUEC_{0\text{-}168h}$. The error bars represent the standard deviations (**P* value < 0.05 vs. placebo; ***P* value < 0.0001 vs. placebo).

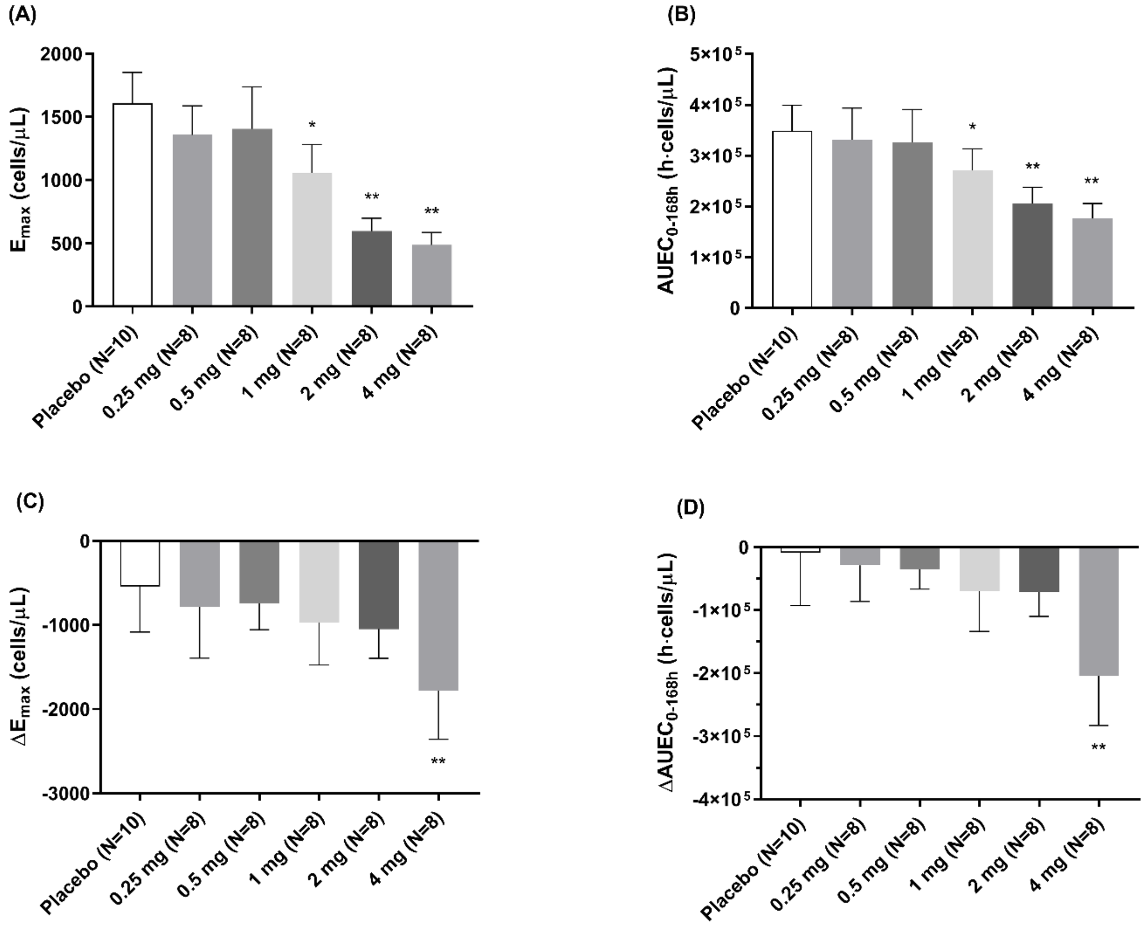

Supplement: Supplementary file 1 — Figure S1 [file CTS-15-1074-s002.pdf]
