# Supplementary material for: Single‐dose of LC51‐0255, a selective S1P1 receptor modulator, showed dose‐dependent and reversible reduction of absolute lymphocyte count in humans
Source: Clin Transl Sci. 2022 Jan 23;15(4):1074–83. doi: 10.1111/cts.13227 (PMC9010277; doi:10.1111/cts.13227)

Figure S2. Relationship between individual pharmacokinetic parameters vs. pharmacodynamic parameters after a single oral administration of LC51-0255 in healthy male subjects. (A) C_{max} vs. ΔE_{max}; (B) C_{max} vs. $\Delta AUEC_{0-168h}$. (○ = 0.25 mg, N = 8; ● = 0.5 mg, N = 8; △ = 1 mg, N = 8; ▼ = 2 mg, N= 8; □ = 4 mg, N =8)

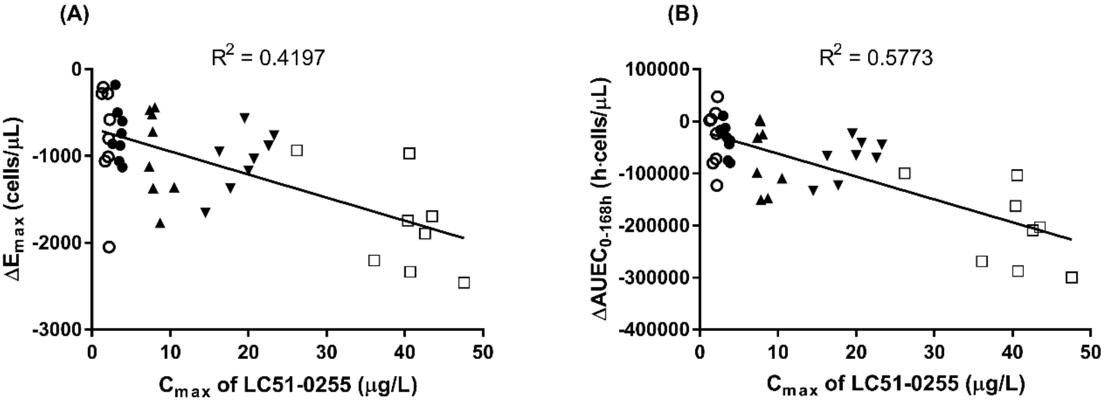

Supplement: Supplementary file 2 — Figure S2 [file CTS-15-1074-s005.pdf]
