# Supplementary material for: Single‐dose of LC51‐0255, a selective S1P1 receptor modulator, showed dose‐dependent and reversible reduction of absolute lymphocyte count in humans
Source: Clin Transl Sci. 2022 Jan 23;15(4):1074–83. doi: 10.1111/cts.13227 (PMC9010277; doi:10.1111/cts.13227)

Figure S3. Relationship between individual plasma concentration of LC51-0255 vs. absolute lymphocyte count change from baseline after a single oral administration of LC51-0255 in healthy male subjects.

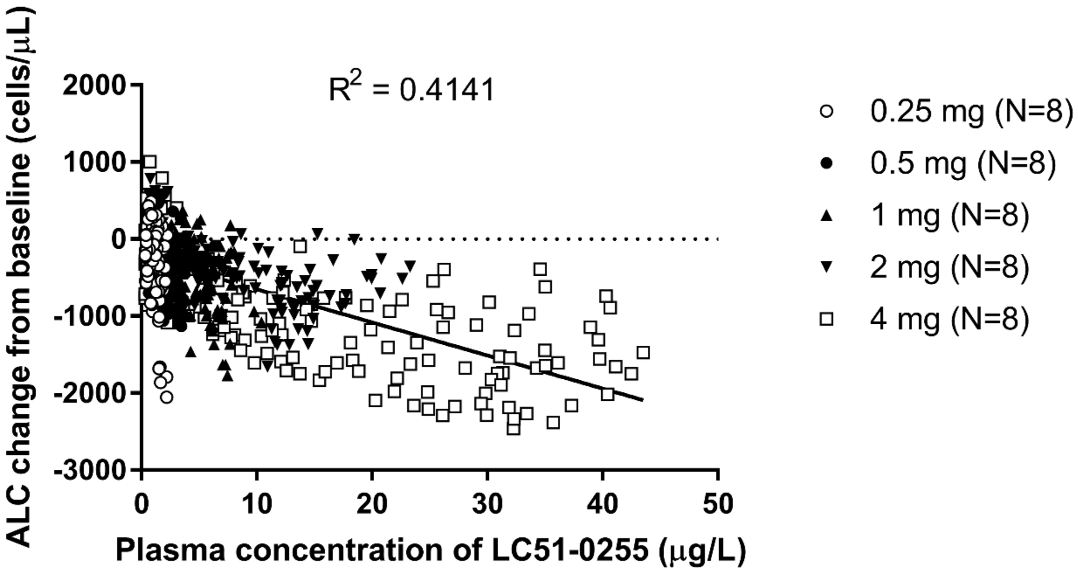

Supplement: Supplementary file 3 — Figure S3 [file CTS-15-1074-s006.pdf]
