# Supplementary material for: Single‐dose of LC51‐0255, a selective S1P1 receptor modulator, showed dose‐dependent and reversible reduction of absolute lymphocyte count in humans
Source: Clin Transl Sci. 2022 Jan 23;15(4):1074–83. doi: 10.1111/cts.13227 (PMC9010277; doi:10.1111/cts.13227)

Figure S4. Time course of absolute lymphocyte count (ALC) and plasma LC51-0255 concentration after a single oral administration of LC51-0255 up to 168 h post-dose in healthy male subjects. Dotted vertical line: time to reach maximum concentration of LC51-0255; Solid vertical line: time to reach maximum ALC reduction. (● = changed from baseline in absolute lymphocyte count; ○ = plasma concentration of LC51-0255)

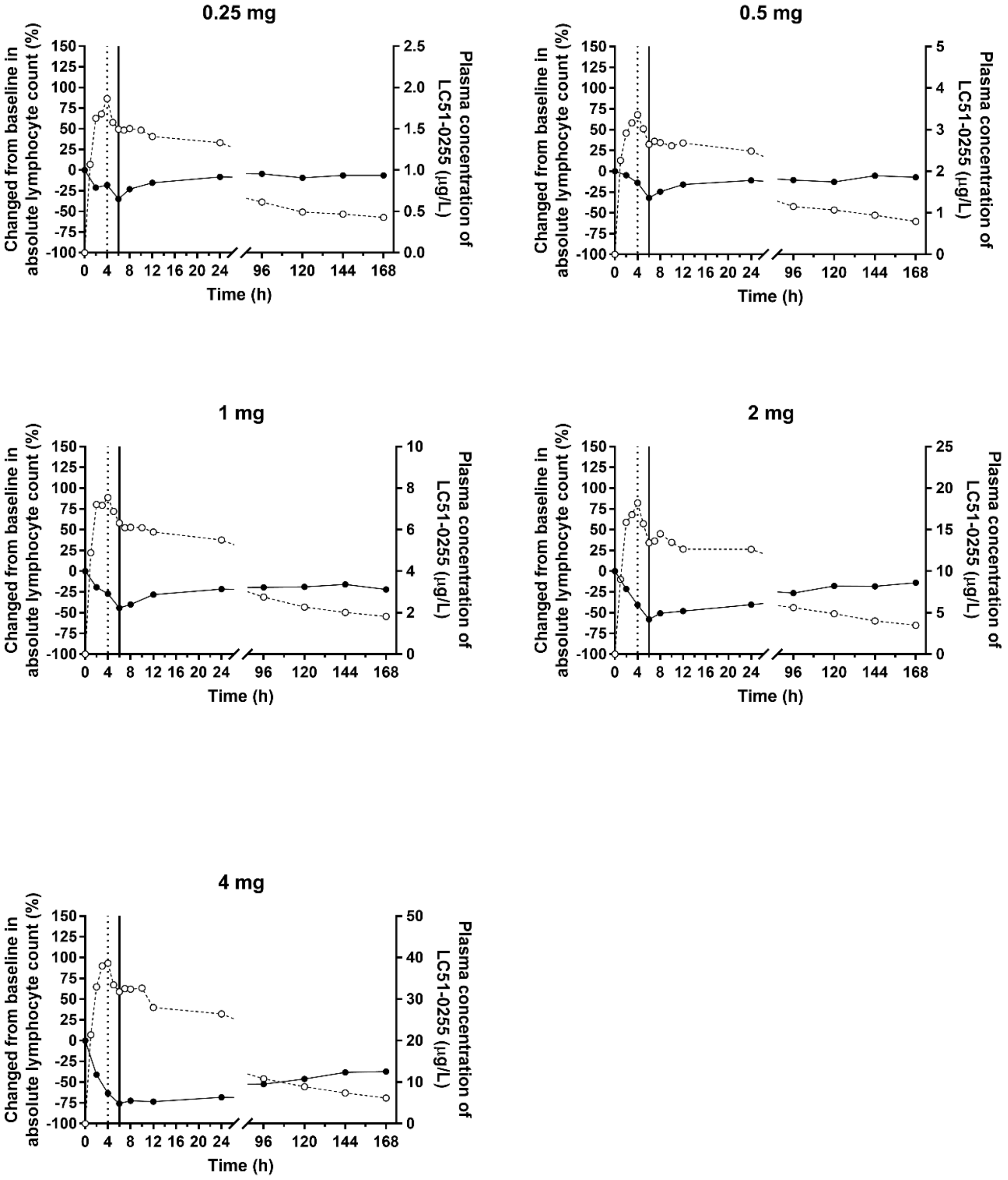

Supplement: Supplementary file 4 — Figure S4 [file CTS-15-1074-s004.pdf]
