# Supplementary material for: Single‐dose of LC51‐0255, a selective S1P1 receptor modulator, showed dose‐dependent and reversible reduction of absolute lymphocyte count in humans
Source: Clin Transl Sci. 2022 Jan 23;15(4):1074–83. doi: 10.1111/cts.13227 (PMC9010277; doi:10.1111/cts.13227)

**Figure S5.** Relationship between plasma concentration of LC51-0255 and changed from baseline in hourly mean heart rate after a single oral administration of LC51-0255 up to 24 hours post-dose. The arrows represent the sequence of measurement.

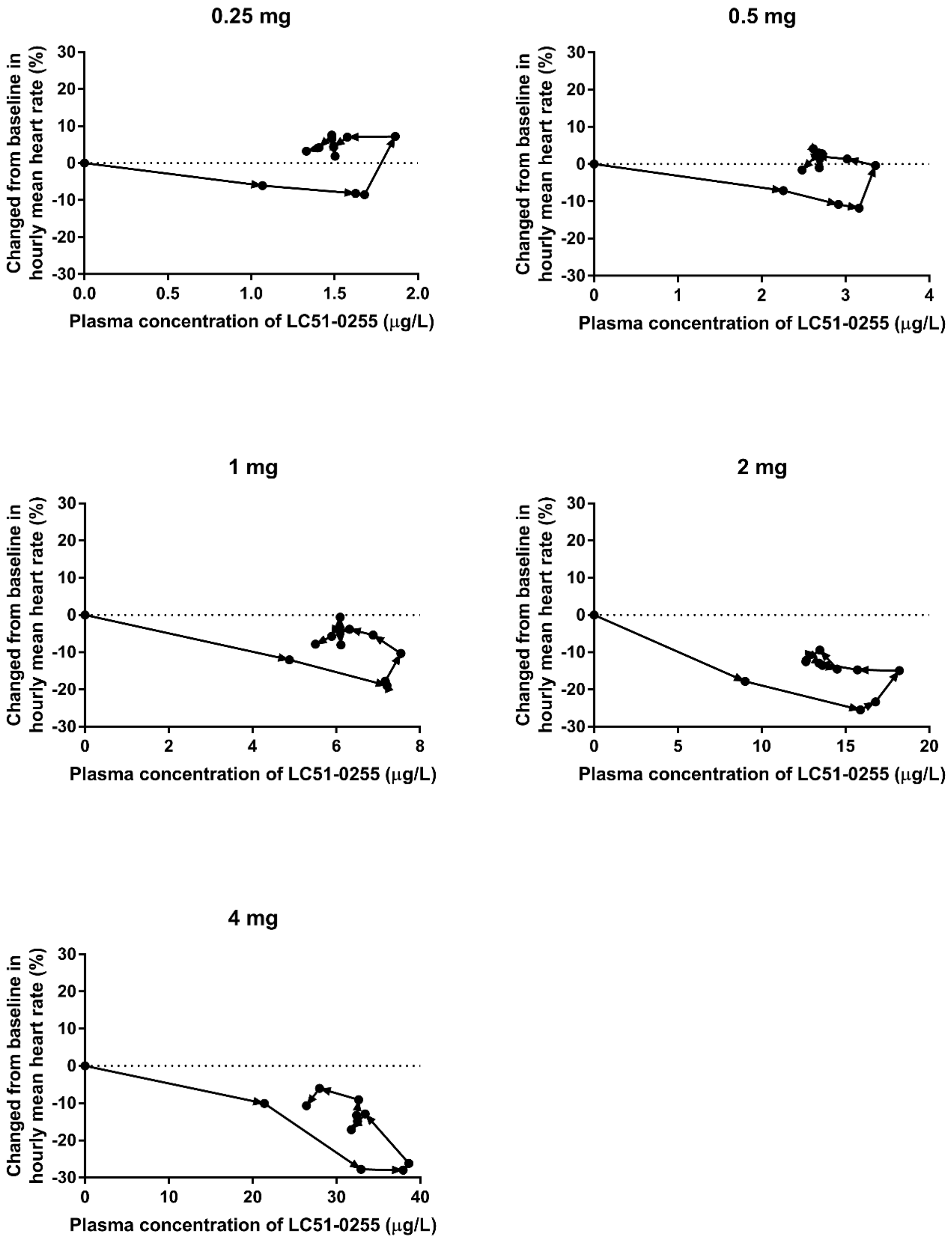

Supplement: Supplementary file 5 — Figure S5 [file CTS-15-1074-s003.pdf]
