# Supplementary material for: Single‐dose of LC51‐0255, a selective S1P1 receptor modulator, showed dose‐dependent and reversible reduction of absolute lymphocyte count in humans
Source: Clin Transl Sci. 2022 Jan 23;15(4):1074–83. doi: 10.1111/cts.13227 (PMC9010277; doi:10.1111/cts.13227)
Supplement: Supplementary file 6 — Table S1 [file CTS-15-1074-s001.docx]

**Table S1. Summary of treatment emergent adverse events by treatment**

|  |  |  |  |  | **LC51-0255** | | | | | |
| --- | --- | --- | --- | --- | --- | --- | --- | --- | --- | --- |
| **System organ class** | | | **Placebo** | | **0.25 mg** | **0.5 mg** | **1 mg** | **2 mg** | **4 mg** | **Total** |
| Preferred term | | | **(N = 10)** | | **(N = 8)** | **(N = 8)** | **(N = 8)** | **(N = 8)** | **(N = 8)** | **(N = 40)** |
|  |  |  |  | |  |  |  |  |  |  |
| **Number of subjects with TEAEs** | | | 0 (0.0%) [0] | | 0 (0.0%) [0] | 1 (12.5%) [1] | 1 (12.5%) [1] | 5 (62.5%) [13] | 6 (75.0%) [18] | 13 (32.5%) [33] |
|  |  |  |  | |  |  |  |  |  |  |
| **Number of subjects with TEAEs that were reported more than one subject** | | | | |  |  |  |  |  |  |
|  | | | | |  |  |  |  |  |  |
| **Cardiac disorders** | | |  | |  |  |  |  |  |  |
| Bradycardia | | | 0 (0.0%) [0] | | 0 (0.0%) [0] | 1 (12.5%) [1] | 0 (0.0%) [0] | 4 (50.0%) [6] | 5 (62.5%) [5] | 10 (25.0%) [11] |
| **Nervous system disorders** | | |  | |  |  |  |  |  |  |
| Dizziness | | | 0 (0.0%) [0] | | 0 (0.0%) [0] | 0 (0.0%) [0] | 0 (0.0%) [0] | 3 (37.5%) [4] | 1 (12.5%) [1] | 4 (10.0%) [5] |
| Headache | | | 0 (0.0%) [0] | | 0 (0.0%) [0] | 0 (0.0%) [0] | 0 (0.0%) [0] | 0 (0.0%) [0] | 2 (25.0%) [3] | 2 (5.0%) [3] |
| **Respiratory thoracic and mediastinal disorders** | | |  | |  |  |  |  |  |  |
| Rhinorrhoea | | | 0 (0.0%) [0] | | 0 (0.0%) [0] | 0 (0.0%) [0] | 0 (0.0%) [0] | 1 (12.5%) [1] | 1 (12.5%) [1] | 2 (5.0%) [2] |

TEAE, treatment emergent adverse events.

The values are displayed as number of subjects (percentage of subjects) [number of events].

Denominator of percentage is the number of subjects in the column.
